# Genetic diversity of *Phytophthora colocasiae* isolates in India based on AFLP analysis

**DOI:** 10.1007/s13205-012-0101-5

**Published:** 2012-12-04

**Authors:** Vishnu Sukumari Nath, Muthukrishnan Senthil, Vinayaka Mahabaleswar Hegde, Muthulekshmi Lajapathy Jeeva, Raj Shekhar Misra, Syamala Swayamvaran Veena, Mithun Raj

**Affiliations:** 1 Division of Crop Protection, Central Tuber Crops Research Institute, Thiruvananthapuram, 695017 Kerala India; 2 Regional Centre of CTCRI, Dumduma HBC P.O, Bhubaneswar, 751 019 Orissa India

**Keywords:** *Phytophthora colocasiae*, AFLP, Spatial structure, Genetic diversity, Disease management

## Abstract

*Phytophthora colocasiae* that causes taro leaf blight is one of the most devastating diseases of taro which is widely distributed in India. Inter and intra-specific genetic diversity among *P. colocasiae* isolates collected from same field was assessed using amplified fragment length polymorphism (AFLP) marker. Seven primer pairs produced 431 markers, of which 428 (99.2 %) were polymorphic. Considerable genetic variability was displayed by the isolates. The average value of the number of observed alleles, the number of effective alleles, mean Nei’s genetic diversity, and Shannon’s information index were 1.993, 1.385, 0.261, and 0.420, respectively. Analysis of molecular variance (AMOVA) showed that the majority (85 %) of the diversity were present within populations of *P. colocasiae*. Dendrogram based on AFLP molecular data using the unweighted pair group method with arithmetic mean (UPGMA) classified the *P*. *colocasiae* isolates into two major clusters irrespective of their geographical origin. Clustering was further supported by principle coordinate analysis. Cophenetic correlation coefficient between dendrogram and original similarity matrix was significant (*r* = 0.816). The results of this study displayed a high level of genetic variation among the isolates irrespective of the geographical origin. The possible mechanisms and implications of this genetic variation are discussed.

## Introduction

Taro ((*Colocasia esculenta* (L.) Schott) is a major root crop of the family Araceae with wide distribution in tropics. It is the fourteenth most consumed vegetable in the world (Lebot and Aradhya 1991). Taro is the fifth most harvested root crops in the world with production estimated at 11.8 million t (Food and Agriculture Organization of the United Nations, 2009). All parts of the plant including corm, cormels, rhizome, stalk, leaves and flowers are edible and prized in various food cultures (Lakhanpaul et al. 2003). The taro plant is a rich source of carbohydrates, proteins, minerals and vitamins (Misra and Sriram 2002) and has medicinal properties to reduce tuberculosis, ulcers, pulmonary congestion and fungal infection (Sharma et al. 2008). Taro corms are utilized in various industries for the preparation of high fructose syrup and alcohols (Misra et al. 2008). These prospects make taro as one of the most important tuber crop.

Leaf blight and corm rot, caused by *Phytophthora colocasiae* is the most destructive disease of taro. The disease affects the leaves and petioles of taro plants, resulting in extensive damage of the foliage. It has become a limiting factor for taro production in all taro growing-countries including India causing yield loss of up to 30–50 % (Jackson et al. 1980; Thankappan 1985; Misra and Chowdhury 1997). Taro leaf blight symptoms appear as small, water-soaked spots, which increase in size and number. With the advancement of the disease, lesions enlarge and become irregular in shape and dark brown in color with yellow margins. Under cloudy weather conditions with intermittent rains and temperature around 28 °C, the disease quickly spreads across entire fields giving them a blighted appearance. Epidemics are favoured by repeated nighttime temperatures close to 20 °C and relative humidity of 90–100 % when zoospore release is greatest (Trujillo 1965; Thankappan 1985). In India, this disease is more prominent in northern and eastern parts, which are potential areas of taro production. In South India, this disease appears occasionally but in serious proportions (Misra and Chowdhury 1997).

Several approaches are used to combat the disease including crop rotation and the use of fungicides. Despite the effectiveness of fungicides the presence of waxy coating on the leaf lamina makes it ineffective, rendering it not economically feasible because large quantities of fungicides and repeated applications are required. Moreover, there are known disadvantages to relying heavily on their use, one being an increased frequency of resistant mutants, especially in pathogen populations with the higher evolutionary potential (McDonald and Linde 2002). Recent years of research have shown an increase in the occurrence and spread of pathogen strains resistant to major types of fungicides and even strains resistant to more than one chemical (Taggart et al. 1999; Robbertse et al. 2001). Furthermore, the use of resistant cultivars is an important method for reducing proliferation of plant pathogens, for this approach to be successful it is essential to analyze the genetic structure of the pathogen populations, as the interplay between resistant cultivars and different pathogen populations is of importance (McDonald and Linde 2002).

Genetic analysis of plant pathogen populations is fundamental to the understanding of the epidemiology, host–pathogen coevolution, and resistance management (Milgroom and Fary 1997). The knowledge of the pathogenic composition of populations is essential for efficient management of taro leaf blight and for initiating suitable breeding programs for the development of resistant cultivars of taro. Despite huge economic loss associated with leaf blight disease, studies reporting genetic diversity analysis in *P. colocasiae* are sparse both globally and at the regional level. Significant genetic diversity in *P. colocasiae* isolates from Southeast Asia and Pacific region has been previously described (Lebot et al. 2003). Variation among *P. colocasiae* isolates in phenotypic characters such as growth rate, colony morphology, metalaxyl resistance and virulence was recognized in old populations (Mishra et al. 2010). Little attention has been paid to genetic diversity analysis within *P. colocasiae* from India, with the exception of one study which reports genetic diversity among 14 *P. colocasiae* isolates using Isozyme and RAPD markers (Mishra et al. 2010). Among several efficient methods for revealing genetic variability within and among *Phytophthora* spp., AFLP technique is the method of choice as it is a robust, reliable molecular marker assay and the number of polymorphisms detected per reaction is much higher than that revealed by restriction fragment linked polymorphisms (RFLP) or the PCR-based randomly amplified polymorphic DNA (RAPD). AFLP has been successfully used for analysis of genetic diversity in *P. infestans* (Abu-El Samen et al. 2003); *P. nemorosa* and *P. pseudosyringae* (Linzer et al. 2009); *P. pinifolia* (Dura’n et al. 2010). To date, no studies have been reported concerning genetic diversity analysis of *P. colocasiae* from the same field.

The objectives of the present study were: (1) to analyze the genetic diversity of *P. colocasiae* populations from same field using AFLP markers (2) to study how this genetic diversity is distributed among and within populations of *P. colocasiae.*

## Materials and methods

### Biological materials

Isolates of *P. colocasiae* used in this study were obtained from same field of taro (*Colocasia esculenta*) showing typical symptoms of leaf blight across different geographical origins of India (Table 1). The regions representing a high disease incidence was given preference for the study.

**Table 1:** Details of *Phytophthora colocasiae* isolates used in this study

Population code	Isolate code	Source	Sample size
Pop 1	AS P1 AS P2 AS P3 AS P4 AS P5 AS P6 AS P7	Assam	7
Pop 2	KE P1 KE P2 KE P3 KE P4 KE P5 KE P6 KE P7 KE P8	Kerala	8
Pop 3	AN P1 AN P2 AN P3 AN P4 AN P5 AN P6	Andhra Pradesh	6
Pop 4	OD P1 OD P2 OD P3 OD P4	Odisha	4

### Isolation of pathogen

For isolation, leaf tissue segments of 1–2 cm from leaf blight infected area were excised. The segments were sterilized in 1 % sodium hypochlorite for 2 min, rinsed twice with sterile distilled water, and placed onto *Phytophthora*-selective media (rye agar amended with 20 mg/L rifamycin, 200 mg/L vancomycin, 200 mg/L ampicillin, 68 mg/L pentachloronitrobenzene, and 50 mg/L 50 % benlate). Hyphae emerging from a diseased tissue were transferred to 2 % water agar. After 3–4 days, single hypha was transferred to a Potato dextrose agar (PDA; 250 g/L potato, 20 g/L dextrose and 20 g/L agar) plate under a dissecting microscope. Each isolate was stored at −20 °C in 50 % glycerol (long-term storage) and at 15 °C on potato dextrose agar (PDA) slants in the dark (short-term storage). The *P. colocasiae* isolates were grown on PDA Petri dishes at 25–28 °C in the dark for mycelium production.

### Genomic DNA isolation

*Phytophthora colocasiae* isolates were grown in Potato dextrose broth medium (PDB; 250 g/L potato, 20 g/L dextrose). For DNA isolation, small blocks (1 cm) of actively growing cultures were used to inoculate Erlenmeyer flasks (250 ml) containing 100 ml of autoclaved potato dextrose broth. The cultures were placed on a rotary shaker (Innova-4230, USA) at 50 rpm and incubated at 28 ± 2 °C. After 5–10 days, depending on the growth of each isolate, mycelia were harvested by filtration through cheesecloth, blotted dry with sterile paper towels, and used immediately for DNA isolation. DNA was isolated using a Genomic DNA purification kit (Fermentas, EU) according to manufacturer’s instructions. The integrity and quality of the DNA isolated were evaluated by electrophoresis on 0.8 % agarose gel using a 1 kb DNA ladder as a DNA size marker.

### AFLP analysis

AFLP analysis was performed as described by Vos et al. (1995) with modifications. Genomic DNA (200 ng) was double digested with 0.5 μL EcoRI (10 U/μl) restriction enzyme at 37 °C for 3 h followed by Taq1 restriction enzyme at 65 °C for another 3 h in a primary 15 μl reaction volume. To the digested DNA was added 10× T4 Buffer (10 mM MgCl_2_, 50 M Tris–HCL, pH 7.5, 10 mM DTT, 1 mM ATP, 25 μg/mL BSA), EcoRI ligation adapter (30 ng) 0.5 μl, Taq I ligation adapter (150 ng) 0.5 μl, T4 DNA ligase (5 U/μl) 0.5 and 3 μl double deionized water, to a final volume of 20 μl. The mixture was incubated at 25 °C overnight. Pre-amplification PCR was performed after diluting the ligated DNA tenfold with double deionized water. A total volume of 25 μl reaction mixture containing 3 μl of the digestion/ligation mixture, 1.0 μl Taq1 primer (200 ng), 1.0 μl EcoRI primer (200 ng), 2.5 μl 10× PCR Buffer (500 mMKCl, 100 mM Tris–HCL, pH 8.3, 15 mM MgCl_2_), 0.5 μl dNTPs (2.5 mM), 0.5 μl Taq DNA polymerase (1 U/μl) and 17.5 μl double deionized water was prepared. PCR reactions were performed with the following cycling parameters: 5 min at 95 °C; 30 cycles of 30 s denaturing at 94 °C, 60 s annealing at 56 °C, and 60 s elongation at 72 °C, ending with 4 °C pause. After checking for the presence of a smear of fragment by 1.5 % agarose electrophoresis, the amplification product was diluted 20 times with double deionized water. After pre-screening 36 primer pairs, 7 selective primer pairs were chosen for this study (Table 2). Each selective AFLP reaction was carried out in a total volume of 25 μl, containing 0.5 μl TaqI selective primer (200 ng), 0.4 μl EcoRI selective primer (200 ng), 2.5 μl 10× PCR Buffer, 0.8 μL dNTPs (2.5 mM), 0.8 μl Taq DNA polymerase (1 U/μl), 2.5 μl pre-amplification products, and 17.5 μl double deionized water. The PCR reactions were performed with the following profile: 2 min at 95 °C; 30 s denaturing at 94 °C, 30 s annealing at 65 °C, and 2 min elongation at 72 °C, followed by reduction of the annealing temperature in each cycle by 0.7 °C for 12 cycles. The annealing temperature was maintained at 56 °C for the remaining 23 cycles. Amplifications were performed in Biorad C1000 thermal cycler (Biorad, Singapore). To the amplification product, an equal volume of formamide loading buffer was added. The amplification product was denatured at 95 °C for 5 min and then electrophoresed on a 6 % denaturing polyacrylamide gel at a constant power of 90 W for approximately 90 min until the forward-running dye reached the end of the gel. AFLP gels were silver stained according to standardized protocol and photographed. Sizes of amplification products were estimated using a 100 bp DNA ladder. All PCRs were repeated at least twice from two different DNA extractions.

**Table 2:** Attributes of AFLP primers used in this study

Marker	Primer	Sequence (5′–3′)	No. of bands scored	No. of polymorphic bands	Mean no. of bands	Polymorphism (%)
1	E + AG/T + AA	CTC GTA GAC TGC GTA CC AG/TACTCAGGACTGGCAA	95	95	30.9	100
2	E + AT/T + AC	CTC GTA GAC TGC GTA CC AT/TACTCAGGACTGGC AC	52	50	18.0	96.1
3	E + AG/T + AT	CTC GTA GAC TGC GTA CC AG/TACTCAGGACTGGC AT	60	59	16.5	98.3
4	E + AC/T + AT	CTC GTA GAC TGC GTA CC AC/TACTCAGGACTGGC AT	40	40	7.2	100
5	E + GC/T + TC	CTC GTA GAC TGC GTA CC GC/TACTCAGGACTGGC TC	77	77	22.0	100
6	E + GA/T + GT	CTC GTA GAC TGC GTA CC GA/TACTCAGGACTGGC GT	51	51	14.2	100
7	E + AC/T + AC	CTC GTA GAC TGC GTA CC AC/TACTCAGGACTGGC AC	56	56	17.7	100
	Total		431	428	126.5	
	Average		61.5	61.1	18.07	99.2

### Data analysis

All clearly detectable AFLP bands were scored for their presence (1) or absence (0) by visual observation. In order to ensure credibility, only reproducible and well-defined bands were scored. Polymorphic and monomorphic bands were determined for each AFLP primer pair, but only polymorphic bands were included in the analysis. Bands were assumed to be independent, and those of identical size were assumed to have identical sequences. A dendrogram was constructed using genetic similarity matrices to display relationships between isolates using the Nei and Li (1979) according to the unweighted pair group mean algorithm using the TREECON software package version 1.3 (Van de Peer 1994). The relative support for the different groups and stability of the dendrogram was assessed by bootstrap analysis (2000 replicates). The cophenetic correlation coefficient was calculated to provide statistical support for the dendrogram obtained, and Mantel’s test (Mantel 1967) was performed to check the goodness-of-fit of the cluster analysis of the matrix on which it was based (1,000 permutations). When the value of a cophenetic correlation coefficient was ≥0.8, this value means that the data within a cluster are most likely to be highly reliable (Mantel 1967). Principal coordinate analysis (PCA) was undertaken for the markers with modules STAND, CORR, and EIGEN of NTSYS-PC using the Euclidean distances derived from the standardized values using the NTSYS-PC-2.2 software.

The similarity matrix was also used to perform a hierarchical analysis of molecular variance (AMOVA) (Excoffier et al. 1992) using FAMD Software version 1.25 (Schluter and Harris 2006). This analysis enables partitioning of the total AFLP variation into within and among geographical region variation components, and provides a measure of inter-region genetic distances as the proportion of the total AFLP variation residing between *P. colocasiae* of any two regions (called Phi statistics).

Allelic frequencies of AFLP marker were used separately to estimate the percentage of polymorphic loci (*P*), mean number of alleles per locus (*A*), effective number of alleles (AE), observed heterozygosity (HO), and expected mean heterozygosity (HE) with respect to Hardy–Weinberg equilibrium (Hedrick 2000) using the computer program POPGENE 32 (Yeh and Yang 1999). Loci were considered polymorphic if more than one allele was detected.

We evaluated the evidence for recombination by performing linkage disequilibrium tests. The standardized index of association (*rBarD*) statistic (Agapow and Burt 2001) was used to estimate linkage disequilibrium (LD) in each population using the software MULTILOCUS version 1.3 (Agapow and Burt 2001). The null hypothesis (*rBarD* = 0) can be rejected when the observed *rBarD* < 0.001, and it can be assumed that the sampled isolates probably originated from a population with a clonal mode of reproduction (Agapow and Burt 2001).

## Results

### Isolation of pathogen

A total of 25 isolates were obtained from several leaf blight infected samples collected from 4 fields of India. Isolation was not successful from decayed or rotten samples. All isolates were positively identified as *P. colocasiae* by comparing their cultural characteristics like colony morphology and sporangial shape with authentic cultures maintained by Central Tuber Crops Research Institute, CTCRI, Thiruvananthapuram. The details of *P. colocasiae* isolates obtained are presented in Table 1.

### AFLP analysis

**Fig. 1:**
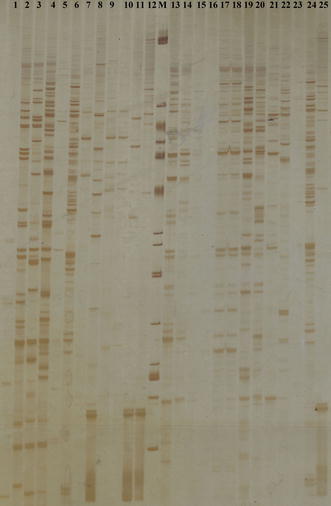
Reprsentative AFLP gel image of 25 isolates of *P. colocasiae* obtained by primer pair E + AG/T + AA. 1–7 (Assam), 8–15 (Kerala), 16–21 (Andhra Pradesh), 22–25 (Odisha)

AFLP analysis produced a large number of reproducible and unambiguous markers for fingerprinting the isolates of *P. colocasiae*. Seven *Eco*RI + 2/*Taq*I + 2 primer pair combinations resolved 431 markers of which 428 (99.2 %) were polymorphic. The bands were distinct and easy to score. There were differences in the numbers of AFLP loci produced by each of the different primer pairs, which presumably reflect differences in sequence composition in the genome. The highest number of amplification products (95) was obtained with the primer pair E + AG/T + AA, while the lowest (51) with E + GA/T + GT pair; the average number of bands among total 7 primer pairs was 61.5. The number of polymorphic fragments detected by each primer varied from 51 to 95, with an average of 61.1. The highest number of polymorphic bands (95) was produced by the primer pair E + AG/T + AA, whereas the primer E + GA/T + GT generated the lowest number of polymorphic bands (51) (Table 2). The amplification pattern with primer pair E + AG/T + AA is shown in Fig. 1.

### Analysis of genetic diversity

**Fig. 2:**
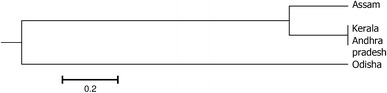
Population genetic tree for four populations of *P. colocasiae* based on Nei’s distance

Population genetic parameters of *P. colocasiae* isolates based on AFLP data are summarized in Table 3. The observed number of alleles (*N*_A_), effective number of alleles (*N*_E_) and Nei’s gene diversity values varied among populations studied. The population distance tree based on Nei’s gene diversity indices is presented in Fig. 2.

**Table 3:** Population genetic parameters of *P. colocasiae* isolates used in this study

Marker	Population code	Polymorphic bands	PPB (%)^a^	*N*_A_^b^	*N*_E_^c^	*H* ^d^	*I* ^e^
AFLP	Assam	351	81.44	1.814 ± 0.389	1.355 ± 0.293	0.229 ± 0.155	0.362 ± 0.218
	Kerala	391	90.72	1.907 ± 0.290	1.403 ± 0.266	0.261 ± 0.138	0.412 ± 0.188
	Andhra Pradesh	372	86.31	1.863 ± 0.344	1.381 ± 0.278	0.247 ± 0.145	0.390 ± 0.201
	Odisha	285	66.13	1.661 ± 0.473	1.315 ± 0.301	0.202 ± 0.167	0.316 ± 0.245
Total		350	81.15	1.993 ± 0.083	1.385 ± 0.219	0.261 ± 0.110	0.420 ± 0.140

^a^Percentage of polymorphic bands (PPB)

^b^Observed number of alleles (*N*_A_)

^c^Effective number of alleles (*N*_E_)

^d^Nei’s gene diversity (*H*)

^e^Shannon’s information index (*I*)

Analysis of molecular variance (AMOVA) based on AFLP data shows that a high percentage of the total genetic diversity of *P. colocasiae* populations in this study was distributed on a small spatial scale with 85 % of the genetic diversity distributed within populations and only 14 % among populations (Table 4). The coefficient of genetic differentiation among populations (*G*_ST_) was 0.091 which supports the AMOVA analysis indicating only limited genetic diversity among populations and significant diversity within populations. The pairwise Φ statistics were 0.147 indicating populations are considerably differentiated. The estimate of gene flow (*Nm*) among populations was 4.97.

**Table 4:** Analysis of molecular variance (AMOVA) for 25 isolates of *P. colocasiae* used in this study

Marker	Source	*df*	SSD	Φ statistics	Variance components	Proportion of variation components (%)
AFLP	Among populations	2	1.33	0.147	0.049	14.71
	Within populations	22	6.31		0.287	85.28
	Total	24	7.65		0.336	

*df* degrees of freedom, *SSD* sums of squared deviations

The observed *rBarD* for four regions viz. Assam, Kerala, Andhra Pradesh and Odisha were 0.0386 (*P* < 0.050), 0.0290 (*P* < 0.100), 0.0203 (*P* < 0.050), 0.0164 (*P* < 0.033), respectively. The result indicates that the *P. colocasiae* populations are a population with a recombination mode of reproduction.

### Cluster analysis

**Fig. 3:**
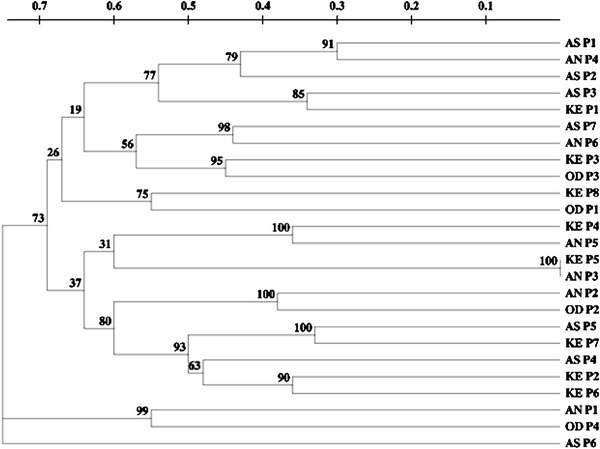
UPGMA dendrogram depicting genetic relationships in 25 isolates of *P. colocasiae* based on AFLP data. Numbers at node represents bootstrap values (2,000 replicates). Isolate codes represent to those mentioned in Table 1 (*AS* Assam, *KE* Kerala, *AN* Andhra Pradesh, *OD* Odisha)

The genetic relationship among 25 isolates of *P. colocasiae* was analyzed by 7 AFLP primer combinations on the basis of Nei and Li distance (Nei and Li 1979). Based on an UPGMA clustering algorithm, the genotypes were grouped into two major clusters (Fig. 3) with high bootstrap values. Cluster I formed the major group in 22 isolates, while cluster II had 3 isolates. Isolates were grouped irrespective of their geographical origin and displayed a high level of genetic diversity among them.

**Fig. 4:**
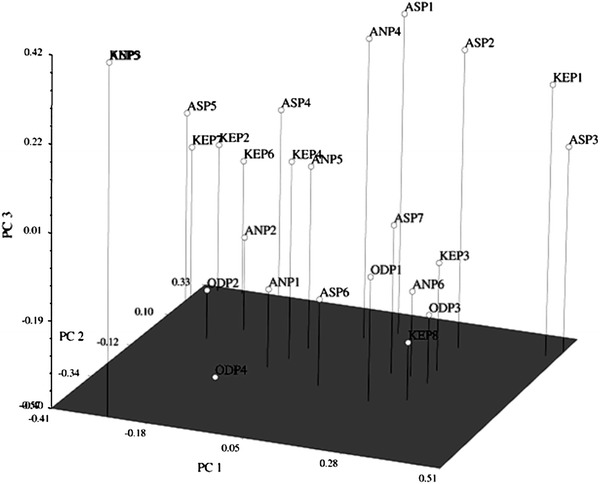
Principal coordinate analysis (PCA) of 25 isolates of *P. colocasiae* based on AFLP data

The cophenetic correlation coefficient between dendrogram and the original similarity matrix was significant (*r* = 0.816) markers supporting a good degree of confidence in the association obtained for 25 isolates of *P. colocasiae*. The results of the PCA derived on the basis of AFLP data illustrated similar trend to cluster analysis. The first three principal coordinate components accounted for 11.91, 20.91 and 29.16 % variation, respectively (Fig. 4).

## Discussion

Pathogens with high levels of genetic diversity, large effective population size, a mixed reproduction system, and high mutation rates are believed to possess the highest evolutionary potential (McDonald and Linde 2002). Information regarding the current *P. colocasiae* population and its evolutionary potential is useful for making informed choice of disease-control strategies to mitigate leaf blight disease. AFLP technique can reveal polymorphisms among closely related isolates to elucidate small changes within a field population over time in response to selection pressures imposed by fungicides/host resistance. AFLP-based genetic markers have been used extensively to assess the genetic diversity and structure of populations of species of *Phytophthora* (Lee et al. 1997; Abu-El Samen et al. 2003; Eikemo et al. 2004).

The work described here is a part of a project aimed at investigating the overall population structure of *P. colocasiae* distributed throughout the Indian subcontinent, in an attempt to explore the possible mechanisms governing their genetic composition. In the present investigation, AFLP technique has been employed to assess the variability of *P. colocasiae* obtained from the same field. Our results demonstrated the utility of AFLP markers to assess genetic diversity among the isolates of *P. colocasiae* from the same field. The high proportion of polymorphic loci found in the isolates revealed profound variability. High levels of genotypic diversity such as those found in the present study have previously been described in *P. colocasiae* populations from Southeast Asia and Pacific region (Lebot et al. 2003) as well as from India (Mishra et al. 2010; Nath et al. 2012).

The dendrogram reconstituted based on the genetic similarity coefficient summarizes the interrelationship among *P. colocasiae* isolates from different geographical locations. The majority of the isolates, irrespective of the geographical origin were clustered together, meaning the genetic distance is not correlated with geographical distance. Confidence limits obtained through bootstrap analysis were high providing strong evidence for the reliability of the clustering of AFLP dataset. The genetic similarity estimates obtained through AFLP analysis displayed profound genetic variation among isolates, 0.25–0.80, respectively. No two isolates depicted close relatedness with each other. For instance, isolates from different fields shared a common clade which supports the fact that migration events are quite common in the population of *P. colocasiae.* A similar observation was reported in previous studies where authors failed to identify geographic grouping in *P. colocasiae* isolates revealed by RAPD and Isozyme markers (Lebot et al. 2003; Mishra et al. 2010). Several studies have reported lack of correlation of geographical origin coupled with molecular marker data (Schilling et al. 1996; Day et al. 2004; Linzer et al. 2009; Cardenas et al. 2011). Even isolates obtained from the same geographical area have different AFLP patterns and were grouped differently, indicating that many populations of this oomycetes are made up of more than one genet and that few are derived clonally. Molecular studies have shown that fungi assumed to be exclusively clonal actually are capable of recombination in nature (Taylor et al. 2000), and this appears to be the case with *P. colocasiae* as well. The presence of larger than expected AFLP variation in isolates of *P. colocasiae* suggests that genetic recombination (or less likely hybridization) is at least possible in this oomycetes.

What is less clear is the cause of the high level of genetic variation in populations of *P. colocasiae*. It is well known that sexual recombination increases genotype diversity in populations, since it creates novel recombinants. Sexually reproducing populations make management of disease more difficult due to the constant appearance of new genotypes that increase the variability of features like fungicide resistance, higher aggressiveness, and better fitness in the population. However, there are several lines of evidence that indicate the absence or rare occurrence of sexual reproduction in *P. colocasiae* as compatible mating types (A1 and A2) are seldom found in the same field (Lin and Ko 2008; Mishra et al. 2010). A high level of recombination is suggested by the low index of association and we speculate that mitotic recombination events greatly contribute to the variation in *P. colocasiae* populations. Alternatively, other mechanisms, such as mutation, translocations, chromosomal deletions and duplications are common in *Phytophthora* species (Goodwin 1997), which may also contribute to genetic variation observed in the *P. colocasiae* populations. Mitotic gene conversion was observed to occur at remarkably high frequencies in *Phytophthora sojae* documenting the potential for rapid generation of variation (Chamnanpunt et al. 2001). Asexual progenies of *P. infestans* have previously been shown to differ from their parents in several characteristics such as aggressiveness, growth rate, colony morphology and virulence (Caten and Jinks 1968; Abu-El Samen et al. 2003). In general, populations with large effective sizes tend to have higher genetic diversity, as more alleles can emerge through mutation and fewer alleles will be lost due to random genetic drift (Hartl and Clark 1997). Variability in the pathogen population also could be elucidated by the fact that the isolates were collected from different climatic classifications, although, the limited number of isolates used in the study would not allow for a robust inference to be made about the influence of climate in the variability in the pathogen population.

For breeding programs aimed at reducing the negative effects of fungal pathogens, the evolutionary potential of populations is of prime importance. Fungal populations are considered to have high evolutionary potential when they have a mixed reproductive system, moderate gene or genotype flow, and large effective population size (McDonald and Linde 2002). The high level of genetic diversity shows that the *P. colocasiae* populations could respond rapidly to selection exerted by newly introduced host resistance genes or fungicides, underlining the importance of relying on integrated disease management. Disease management programmes should focus on local scale than on a regional level since it is likely that effective packages at one particular location may not prove so in other regions. Even though this study does not provide information on the effective population size and only limited information on gene flow, it still provides important evidence on the evolutionary potential of the Indian *P. colocasiae* populations. The small sample sizes in this study, however, restrict the relevance of the analysis and the credibility of results for more generalized conclusions. Further studies should therefore be carried out, using larger populations from more extended geographical regions to gain insights into the origin of diversity in this important plant pathogen.
